# UHRF1 is a novel diagnostic marker of lung cancer

**DOI:** 10.1038/sj.bjc.6605717

**Published:** 2010-06-01

**Authors:** M Unoki, Y Daigo, J Koinuma, E Tsuchiya, R Hamamoto, Y Nakamura

**Affiliations:** 1 Laboratory for Biomarker, The Institute of Physical and Chemical Research, Center for Genomic Medicine, RIKEN, Tokyo 108-8639, Japan; 2 Laboratory of Molecular Medicine, Human Genome Center, Institute of Medical Science, The University of Tokyo, Tokyo 108-8639, Japan; 3 Department of Pathology, Saitama Cancer Center, Saitama 362-0806, Japan; 4 Molecular Pathology and Genetics Division, Kanagawa Cancer Center Research Institute, Kanagawa 241-0815, Japan

**Keywords:** UHRF1, lung cancer, adenocarcinoma, squamous cell carcinoma, diagnostic marker

## Abstract

**Background:**

Lung cancer is the leading cause of cancer deaths worldwide. As the sensitivity and specificity of current diagnostic markers are not perfect, we examined whether ubiquitin-like with PHD and ring finger domains 1 (UHRF1), which is overexpressed in various cancers but not yet examined in lung cancer in large scale, can be a novel diagnostic marker of lung cancer.

**Methods:**

Immunohistochemical analysis using surgical specimens obtained from 56 US and 322 Japanese patients with lung cancer was performed.

**Results:**

The UHRF1 was stained specifically in the nuclei of cancer cells, but not in the other cells. The UHRF1 expression was observed in all histological types of lung cancer, especially in non-adenocarcinomas (non-ADCs), both in the US and Japanese cases. In 322 Japanese non-small cell lung cancer (NSCLC) cases, UHRF1 expression was associated with the histological type (higher in non-ADCs; *P*<0.00001), gender (higher in male; *P*=0.00082), smoking (higher in smokers; *P*=0.00004), pT factor (higher in advanced stage; *P*=0.00010), and pN factor (higher in cancers with metastasis in regional lymph nodes; *P*=0.00018). The UHRF1 expression was also associated with poor prognosis for NSCLC patients (*P*=0.0364). Although UHRF1 overexpression was associated with these malignant indicators, UHRF1 was detectable in half of lung cancer patients in an early pathological stage.

**Conclusion:**

The UHRF1 is overexpressed in various types of lung cancer from early pathological stage. Therefore, detection of UHRF1 expression in tissue specimens by immunohistochemistry can be useful for diagnosis of lung cancer in all pathological stages.

Lung cancer is the leading cause of cancer deaths worldwide including the United States and Japan according to the World Health Organization (WHO) database (http://www.who.int/en/). Among various histological types of lung cancers, non-small cell lung cancer (NSCLC) accounts for approximately 80%, whereas small cell lung cancer (SCLC) accounts for approximately 15%. The two major histological types of NSCLC are adenocarcinoma (ADC) and squamous cell carcinoma (SCC). In Japan, SCC accounts for 34.7% and 16.3% of all lung cancer cases in males and females, respectively. On the other hand, ADC accounts for 42.9% and 67.2% of all lung cancer cases in males and females, respectively (incident year 1999–2003; Toyoda *et al*, 2008). The remaining few per cent of NSCLC are other histological types of carcinomas including large cell carcinoma and adenosquamous cell carcinoma; SCC is more common in males, and smoking dramatically increases the risk of this type of cancer; the relative risks of smoking in males were 11.7 and 2.3 for SCC and ADC, respectively, and were 11.3 and 1.4 correspondingly in females in Japan (Wakai *et al*, 2006). Smoking rates of Japanese males and females in 2007 are 43.3% and 12.0%, respectively, according to Japanese exposure factors handbook published by National Institute of Advanced Industrial Science and Technology in Japan (http://unit.aist.go.jp/riss/crm/exposurefactors/english_summary.html). The 5-year survival rate based on pathologic (p) stages was analysed using a large population of Japanese lung cancer cases (*n*=13 010) in 2005 (Asamura *et al*, 2008). According to the study, the 5-year survival rates of SCLC patients who underwent plumonary resections were as follows: 58.3%, 60.2%, 40.6%, 41.1%, 28.3%, 34.6%, and 30.8% for IA, IB, IIA, IIB, IIIA, IIIB, and IV, respectively. The 5-year survival rates of NSCLC patients who underwent plumonary resections were as follows: 83.9%, 66.3%, 61.0%, 47.4%, 32.8%, 29.6%, and 23.1% for IA, IB, IIA, IIB, IIIA, IIIB, and IV, respectively. This study also showed that ADC histological type, female gender, and age <50 years were significant favourable prognostic factors.

Although there are several biomarkers in clinical use such as SCC antigen, carcinoembryonic antigen, neuron-specific enolase, and pro-gastrin-releasing peptide, none of them are perfect in terms of sensitivity and/or specificity (Ando *et al*, 2003; Molina *et al*, 2005). Thus, we have been seeking additional sensitive and cancer-specific biomarkers detectable in serum and tumour tissues to improve the current high mortality of lung cancer, and found more than 20 candidate diagnostic and/or prognostic biomarkers, which are now under development for clinical use (Daigo and Nakamura, 2008). As many molecular signalling pathways are disrupted during lung carcinogenesis, we believe that combinations of biomarkers are necessary for precise diagnosis and prognosis of lung cancer, and thus we continue to search for superior biomarkers.

Ubiquitin-like with PHD and ring finger domains 1 (UHRF1), also known as ICBP90, was identified as a protein whose expression is only detectable in proliferating cells, not in quiescent cells (Hopfner *et al*, 2000; Unoki *et al*, 2004). Recently, it was revealed that UHRF1 has a central function in epigenetic modulation during DNA duplication in the S phase (Sharif *et al*, 2007; Arita *et al*, 2008; Avvakumov *et al*, 2008; Hashimoto *et al*, 2008). Up-regulation of UHRF1 has been reported in various cancers (Mousli *et al*, 2003; Crnogorac-Jurcevic *et al*, 2005; Jenkins *et al*, 2005; Lorenzato *et al*, 2005; Oba-Shinjo *et al*, 2005; Pita *et al*, 2009; Unoki *et al*, 2009). As no large-scale study of UHRF1 expression in lung cancers has been performed, we examined whether UHRF1 could be a novel diagnostic marker of lung cancer by immunohistochemical analysis to understand the clinical importance of this protein in lung carcinogenesis.

In this report, we examined UHRF1 expression using 56 US and 322 Japanese lung cancer cases by immunohistochemical analysis and found that expression of UHRF1 was significantly up-regulated in almost all histological types of lung cancers, especially in non-ADCs. The expression of UHRF1 was associated with poor prognosis and several other clinicopathological characteristics of the lung cancer patients. As UHRF1 was specifically expressed in cancer cells and detectable in approximately half of lung cancer cases in an early pathological stage, UHRF1 could be a novel diagnostic marker for lung cancer.

## Materials and methods

### Lung cancer clinical tissue specimens

A total of 56 formalin-fixed primary lung cancer tissues from US patients including SCLC and NSCLC was purchased from BioChain Institute (Table 1; Cat T8235724-5, lot B207162; Cat T2235152, lot A504256; Cat T2235152-9, lot A604318, Hayward, CA, USA). A total of 322 formalin-fixed primary Japanese NSCLCs (Table 2) and adjacent normal lung tissue samples used for immunostaining on tissue microarrays was obtained from patients undergoing curative surgical operation at Saitama Cancer Center (Saitama, Japan) (Ishikawa *et al*, 2004, 2007). A total of 23 frozen primary lung cancer tissues for RNA extraction was obtained as published earlier (Kikuchi *et al*, 2003; Taniwaki *et al*, 2006). This study as well as the use of all clinical materials described above was approved by individual institutional Ethical Committees (Ishikawa *et al*, 2004, 2007). Histological classification of tumours was performed based on the WHO criteria. All tumours were staged based on the pathological tumour-node-metastasis classification of the International Union Against Cancer. Histopathological examination of resected tumours revealed that 192 cases were diagnosed as ADC, and the other 130 cases were classified as non-adenocarcinoma (non-ADC).

### RNA extraction and qRT–PCRs

Total RNA was extracted from frozen clinical tissues using the TRIzol reagent (Life Technologies, Inc., Gaithersburg, MD, USA) according to the manufacturer’s protocol. Extracted RNAs were treated with DNase I (Nippon Gene) and reversely transcribed using oligo(dT) primer and SuperScript II (Invitrogen, Tokyo, Japan). For real-time TaqMan qRT–PCRs, specific primers and probes, which strictly amplify only cDNA and not genomic DNA, for human *UHRF1* and *β2-microglobulin* were purchased from Applied Biosystems (Carlsbad, CA, USA; ID: Hs00273589_m1 and 4333766F, respectively). The PCRs were performed using the ABI Prism 7700 Sequence Detection System (Applied Biosystems) following the manufacturer's protocol. Amplification conditions were 2 min at 50°C, 10 min at 95°C, and then 40 cycles each consisting of 15 s at 95°C and 1 min at 60°C. The CT value obtained by *UHRF1* amplification was compared among the samples after normalisation using *β2-microglobulin* expression levels as an endogenous control.

### siRNA experiments

The SBC-5 cells, whose origin was SCLC, were obtained from Japanese Collection of Research Bioresources (Osaka, Japan). The cell line was grown in a monolayer in Eagle's Minimum Essential Medium supplemented with 10% foetal bovine serum, penicillin/streptomycin, and glutamine, at 37°C in 5% CO_2_. siRNA oligonucleotide duplexes were purchased from SIGMA Genosys (Sigma Aldrich Japan, Tokyo, Japan) for targeting the human *UHRF1* transcript or the *EGFP* and *FFluc* transcripts. The siRNA targeting sequences (sense strand) are as follows: *UHRF1*, 5′-CUGCUUUGCUCCCAUCAAU-3′ *EGFP*, 5′-GCAGCACGACUUCUUCAAGTT-3′ *FFluc*, 5′-GUGCGCUGCUGGUGCCAACTT-3′. The SBC-5 cells were transfected with *EGFP* siRNA, *FFluc* siRNA, or the two different *UHRF1* siRNAs at a final concentration of 20 nM using Lipofectamine 2000 reagent (Invitrogen) according to the manufacturer's protocol. The siRNA transfection efficiency under the condition was 100%, as evaluated by a fluorescent siRNA (data not shown). Then the cells were harvested after 48 h of transfection for western blotting analysis. Western blotting was performed using anti-UHRF1 mouse monoclonal antibody (1 : 1000, BD Bioscience, Tokyo, Japan) or anti-*β*-actin mouse monoclonal AC-15 (Sigma Aldrich Japan).

### Tissue microarray construction

Lung cancer tissue microarrays were constructed as published earlier using formalin-fixed lung cancer tissues (Ishikawa *et al*, 2004; Furukawa *et al*, 2005; Kato *et al*, 2005). Tissue areas for sampling were selected based on visual alignment with the corresponding haematoxylin and eosin-stained sections on slides. Three to five tissue cores (diameter=0.6 mm; height=3–4 mm), which were taken from donor tumour blocks, were placed into recipient paraffin blocks using a tissue microarrayer (Beecher Instruments, Sun Prairie, WI, USA). A core of normal tissue area was punched from each case. A total of 5-*μ*m sections of the resulting microarray block were used for immunohistochemical analysis.

### Immunohistochemical staining analysis

The expression patterns of UHRF1 in lung cancer and normal human lungs were examined by immunohistochemistry as described earlier (Unoki *et al*, 2004, 2009). Briefly, slides of paraffin-embedded lung tumour specimens were processed under high pressure (125°C, 30 s) in antigen-retrieval solution with high pH (pH 9, Dako Cytomation, Carpinteria, CA, USA), treated with peroxidase blocking regent, and then treated with protein blocking regent (K130, X0909, Dako Cytomation). Tissue sections were incubated with anti-UHRF1 mouse monoclonal antibody (1 : 1000, BD Bioscience), or normal mouse IgG (1 : 100, Santa Cruz Biotechnology, Santa Cruz, CA, USA) followed by HRP-conjugated secondary antibody (Dako Cytomation). Antigen was visualised with substrate chromogen (Dako liquid DAB chromogen; Dako Cytomation). Finally, tissue specimens were stained with Mayer’s haematoxylin (Muto pure chemicals Ltd., Tokyo, Japan) for 1 min to discriminate the nucleus from the cytoplasm.

### Statistical analysis

A *χ*^2^ test was applied for evaluating associations between UHRF1 immunoreactivity and clinicopathological characteristics of patients. Tumour-specific survival curves were calculated from the date of surgery to the time of death related to lung cancer, or to the last follow-up observation. The Kaplan–Meier method was applied to generate the survival curves. Survival differences were analysed with the log-rank test based on the status of UHRF1 expression. This analysis was performed using StatView (version 5.0; SAS Institute, Inc., Cary, NC, USA).

## Results

### UHRF1 was highly expressed in the US lung cancer cases

**Figure 1:**
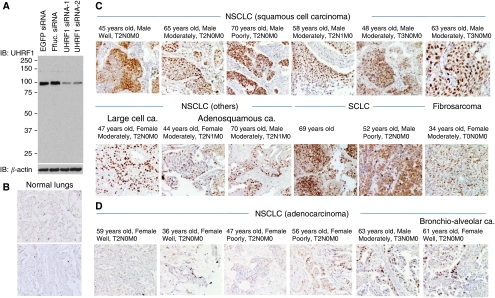
Expression of UHRF1 in the US lung cancer cases detected by immunohistochemistry. (**A**) Specificity of the anti-UHRF1 antibody used for immunohistochemical analysis. The SBC5 cells were transfected with *EGFP* siRNA, FFluc siRNA, or two independent siRNAs targeting *UHRF1* mRNA. Cells were harvested after 48 h of transfection, and endogenous UHRF1 was detected by western blotting using the anti-UHRF1 antibody. *β*-actin was used as a loading control. (**B**) Representative data of UHRF1 staining in adjacent normal lungs of US samples ( × 200). (**C**) Representative data of UHRF1 staining in SCLC, fibrosarcoma, and non-ADC histological types of NSCLC including SCC, large cell carcinoma, and adenosquamous carcinoma ( × 200). (**D**) Representative data of ADC including bronchio-alveolar carcinoma ( × 200).

We screened in-house cDNA microarray database (Kikuchi *et al*, 2003; Taniwaki *et al*, 2006), and found that *UHRF1* mRNA was overexpressed in 67% of NSCLCs and in 93% of SCLCs compared with their adjacent normal lungs. To validate the microarray data, we examined UHRF1 protein expression levels in the 56 US lung cancer cases by immunohistochemistry with information of age, gender, histological type, and pT and pN factors of their cancers (Table 1). First we evaluated specificity of an anti-UHRF1 antibody by western blotting using cellular lysate from SBC5 cells transfected with two control siRNAs and two UHRF1 siRNAs. The result revealed that the antibody specifically recognises endogenous UHRF1 (Figure 1A). Using the antibody, we performed immunohistochemistric analysis. The analysis revealed that UHRF1 was not expressed in adjacent normal lungs, stromal cells, and invaded inflammatory cells, but was specifically expressed in the nuclei of cancer cells (Figure 1B–D). Normal mouse IgG served as a negative control of primary antibody in each case, and no staining was observed (data not shown). We scored the staining levels of UHRF1 as high or low. The UHRF1 was overexpressed in 66% of the overall NSCLCs (Table 1). Interestingly, although expression of UHRF1 was detected in almost all histological types of the lung cancers, its expression was significantly higher in non-ADCs (Table 1); 84% of non-ADCs showed high expression of UHRF1, whereas 32% of ADCs were overexpressed UHRF1 (*P*=9 × 10^−5^, *χ*^2^ test). Other clinicopathlogical factors did not show any correlation with UHRF1 expression levels in the US samples. Importantly, UHRF1 expression was observed in 50% of lung cancer cases in the early stage (T0+T1) (Table 1).

### Overexpression of UHRF1 was observed in Japanese lung cancer cases and associated with histological type, smoking, and gender

**Figure 2:**
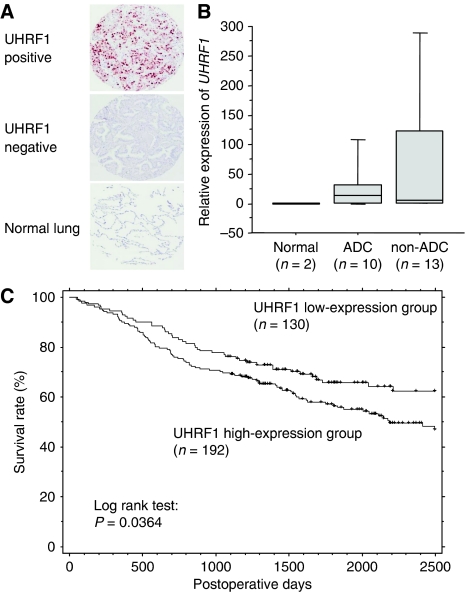
Expression of UHRF1 in the Japanese lung cancer cases detected by immunohistochemistry. (**A**) Representative examples of high (positive) and low (negative) expression of UHRF1 in lung cancers and normal lungs ( × 100). (**B**) Expression levels of *UHRF1* mRNA in Japanese lung cancer cases were measured by TaqMan qRT–PCR. *β2-microgloblin* was used for internal control. The *UHRF1* mRNA was up-regulated in lung cancers, especially in non-adenocarcinoma (non-ADC). (**C**) Kaplan–Meier analysis of tumour-specific survival in lung cancer patients according to UHRF1 expression levels. The UHRF1-high-expression group (*n*=192) showed significantly shorter survival periods compared with the low-expression group (*n*=130) (*P*=0.0364: log-rank test).

As the number of samples of the US cases was relatively small, we analysed an additional 322 Japanese lung cancer cases with information of postoperative clinical course, pT and pN factors of their cancers, and smoking habit, besides information of age, gender, and histological type (Table 2). We performed immunohistochemical analysis and scored the staining levels of UHRF1 as high or low similarly to the US cases (Figure 2A). Overexpression of UHRF1 was observed in 60% of the overall NSCLC cases (Table 2). A *χ*^2^ test revealed that UHRF1 overexpression was strongly associated with histological type of the Japanese cases (higher in non-ADC; *P*=4.2 × 10^−11^). The UHRF1 high expression was also associated with smoking (higher in smokers; *P*=0.00004 by *χ*^2^ test) and gender (higher in males; *P*=0.00082 by *χ*^2^ test) (Table 2). Among these three factors, the histological type showed the strongest correlation with overexpression of UHRF1 (*P*=4.2 × 10^−11^). We also detected *UHRF1* mRNA in Japanese cases by quantitative TaqMan PCR and found that *UHRF1* mRNA was up-regulated in the overall lung cancers, especially in non-ADC, as the same as UHRF1 protein detected by immunohistochemistry (Figure 2B). At mRNA level, overexpression of *UHRF1* was observed in the overall NSCLS cases even in ADC; *UHRF1* mRNA levels were increased in 60% of ADC and in 77% of non-ADC.

### Overexpression of UHRF1 was associated with lung cancer malignancy

Similar to the other cancers (Oba-Shinjo *et al*, 2005; Unoki *et al*, 2009), UHRF1 expression was associated with the malignant nature of lung cancer in Japanese cases (Table 2); UHRF1 high expression was significantly correlated with pT factor (higher in advanced stage; *P*=0.00010 by *χ*^2^ test) and pN factor (higher in cancers with metastasis in regional lymph nodes; *P*=0.00018 by *χ*^2^ test). Although overexpression of UHRF1 associated with lung cancer malignancy, approximately half (47%) of Japanese lung cancer patients in T1 stage showed high expression of UHRF1 similarly to the US cases (Table 2). Kaplan–Meier analysis revealed that the high expression of UHRF1 was associated with poor prognosis (*P*=0.0364 by log-rank test) (Figure 2C).

## Discussion

Lung cancer is the most common cancer in terms of both incidence and mortality worldwide (1.35 million new cases per year and 1.18 million deaths) according to Cancer Research UK. Survival rate of the cancer is still poor, partially because more than two-thirds of lung cancers are diagnosed at a late stage. Therefore, diagnostic markers of lung cancer, which can detect early stage lung cancer, are required to improve the current situation. Sensitivity and/or specificity of current diagnostic markers still need to be improved (Ando *et al*, 2003; Molina *et al*, 2005). We have been seeking superior diagnostic markers and identified a couple dozen of candidates (Daigo and Nakamura, 2008). However, we are still trying to identify other diagnostic markers to increase accuracy of current diagnosis for responding to individual lung cancers, which are caused by different genetic and epigenetic alterations.

The UHRF1 is significantly overexpressed in various cancers (Mousli *et al*, 2003; Crnogorac-Jurcevic *et al*, 2005; Jenkins *et al*, 2005; Lorenzato *et al*, 2005; Oba-Shinjo *et al*, 2005; Pita *et al*, 2009; Unoki *et al*, 2009). The UHRF1 transmits methylation status from mother cells to daughter cells by recognising hemi-methylated DNA and by recruiting DNMT1 to methylate both DNA strands (Sharif *et al*, 2007; Arita *et al*, 2008; Avvakumov *et al*, 2008; Hashimoto *et al*, 2008). The UHRF1 also recognises trimethyl histone H3 lysine 9 and recruits histone modification enzymes such as histone deacetylase 1 (HDAC1), a methyltransferase G9a, and a histone acetylase Tip60 (Unoki *et al*, 2004; Karagianni *et al*, 2008; Achour *et al*, 2009; Kim *et al*, 2009). Thus, UHRF1 has a central function in epigenetic transcriptional regulation. It has been suggested that UHRF1 localises on methylated promoters of tumour suppressor genes and suppresses expression of these genes with transcriptional repressors such as G9a and HDAC1 (Unoki *et al*, 2004; Jin *et al*, 2009). These results indicate that UHRF1 is fundamentally important for cell proliferation.

In this report, we performed, for the first time, a large-scale analysis of UHRF1 expression in lung cancer cases with clinical information. We observed overexpression of UHRF1 in lung cancer cases, especially in non-ADC cases, regardless of ethnic groups, indicating that frequent overexpression of UHRF1 in non-ADC is common worldwide. Although UHRF1 is fundamentally important for cell proliferation, UHRF1 ‘low-expression’ population exists. The qRT–PCR, which is more sensitive than immunohistochemistry, revealed that *UHRF1* mRNA level was up-regulated in approximately 70% of the overall lung cancer cases. In 30% of the rest of lung cancers, other oncogenic pathways may be predominantly activated. We further found that overexpression of UHRF1 in Japanese lung cancer cases associated with gender (higher in male) and smoking. The major histological type of non-ADCs is SCC, which is more common in males (Toyoda *et al*, 2008), and smoking is the biggest risk factor for SCC (Wakai *et al*, 2006). In Japan, the smoking ratio for males is approximately four-fold higher than that of females. Thus, it is reasonable that UHRF1 overexpression associated with these three factors, the histological type, gender, and smoking, although further analysis is required for clarifying biological functions of UHRF1 in carcinogenesis of non-ADCs. In the United States, prevalence of smoking was also higher among males than females (23.9% and 18%, respectively, in 2006) according to the Centers for Disease Control and prevention (http://www.cdc.gov/), but the difference between the genders in the United States is smaller than that in Japan (1.3-fold *vs* 4-fold). We think that this fact reflected our result that correlation between gender and UHRF1 expression levels in the US cases was not significant.

Overexpression of UHRF1 also associated with cancer malignancy indicated by pT and pN factors and survival rate in Japanese cases. No correlation between UHRF1 expression and pT-pN factors was observed in the US cases because of a lack of statistical power. However, we would like to emphasise that overexpressed UHRF1 was detectable in approximately half of lung cancer patients in an early pathological stage by immunohistochemistry, indicating that UHRF1 can be a diagnostic marker of lung cancer even in the early stage. Combinations of UHRF1 and several other lung cancer biomarkers including ones originally identified by our group (Daigo and Nakamura, 2008) may yield a better outcome. In conclusion, our data indicate that immunohistochemical staining of UHRF1 may ameliorate the sensitivity and specificity of current sputum examination for cancer detection, and/or to score the malignant potential of lung cancer using biopsy/resected tissue specimens accurately.

## Figures and Tables

**Table 1:** Association between the patients’ characteristics and UHRF1 expression in 56 US lung cancer cases

	**UHRF1 expression**			
	**High (*n*=37: 66%)**	**Low (*n*=19: 34%)**	**Total (*n*=56: 100%)**	***P*-value (*χ*^2^ test)**
*Age (years)*				
<65	15 (62%)	9 (38%)	24 (100%)	0.625
⩾65	22 (69%)	10 (31%)	32 (100%)	
				
*Gender*				
Female	10 (56%)	8 (44%)	18 (100%)	0.253
Male	27 (71%)	11 (29%)	38 (100%)	
				
*Histological type*				
ADC	6 (32%)	13 (68%)	19 (100%)	0.00009^*^
Non-ADC	31 (84%)	6 (16%)	37 (100%)	
				
*pT factor*				
T0+T1	5 (50%)	5 (50%)	10 (100%)	0.236
T2+T3	32 (70%)	14 (30%)	46 (100%)	
				
*pN factor*				
N0	30 (65%)	16 (35%)	46 (100%)	0.772
N1	7 (70%)	3 (30%)	10 (100%)	

Abbreviations: ADC=adenocarcinoma; non-ADC=non-adenocarcinoma; UHRF1=ubiquitin-like with PHD and ring finger domains 1.

^*^Statistically significant.

**Table 2:** Association between the patients’ characteristics and UHRF1 expression in 322 Japanese NSCLC cases

	**UHRF1 expression**			
	**High (*n*=192: 60%)**	**Low (*n*=130: 40%)**	**Total (*n*=322: 100%)**	***P*-value (*χ*^2^ test)**
*Age (years)*				
<65	90 (61%)	72 (49%)	148 (100%)	0.13402
⩾65	102 (59%)	58 (41%)	174 (100%)	
				
*Gender*				
Female	46 (46%)	54 (54%)	100 (100%)	0.00082^*^
Male	146 (66%)	76 (34%)	222 (100%)	
				
*Histological type*				
ADC	86 (45%)	106 (55%)	192 (100%)	4.2 × 10^−11^^**^
Non-ADC	106 (82%)	24 (18%)	130 (100%)	
				
*pT factor*				
T1	63 (47%)	71 (53%)	134 (100%)	0.00010^*^
T2+T3	129 (69%)	59 (31%)	188 (100%)	
				
*pN factor*				
N0	107 (52%)	99 (48%)	206 (100%)	0.00018^*^
N1+N2	85 (73%)	31 (27%)	116 (100%)	
				
*Smoking*				
Never	38 (42%)	53 (58%)	91 (100%)	0.00004^*^
Smoking	154 (67%)	77 (33%)	231 (100%)	

Abbreviations: ADC=adenocarcinoma; non-ADC=non-adenocarcinoma; NSCLC=non-small cell lung cancer; UHRF1=ubiquitin-like with PHD and ring finger domains 1.

^*^Statistically significant.

^**^Statistically most significant.
